# Primary Human Hepatocyte Spheroids as Tools to Study the Hepatotoxic Potential of Non-Pharmaceutical Chemicals

**DOI:** 10.3390/ijms222011005

**Published:** 2021-10-12

**Authors:** Vânia Vilas-Boas, Eva Gijbels, Kaat Leroy, Alanah Pieters, Audrey Baze, Céline Parmentier, Mathieu Vinken

**Affiliations:** 1 Entity of In Vitro Toxicology and Dermato-Cosmetology, Vrije Universiteit Brussel, 1090 Brussels, Belgium; vfevilasboas@gmail.com (V.V.-B.); eva.anne.gijbels@vub.be (E.G.); kaat.leroy@vub.be (K.L.); alanah.pieters@vub.be (A.P.); 2 KaLy-Cell, 67115 Plobsheim, France; a.baze@kaly-cell.com (A.B.); c.parmentier@kaly-cell.com (C.P.)

**Keywords:** primary human hepatocytes, spheroids, cholestasis, hepatotoxicity, cyclosporine-A, bosentan, macitentan, paraquat, tartrazine, triclosan

## Abstract

Drug-induced liver injury, including cholestasis, is an important clinical issue and economic burden for pharmaceutical industry and healthcare systems. However, human-relevant in vitro information on the ability of other types of chemicals to induce cholestatic hepatotoxicity is lacking. This work aimed at investigating the cholestatic potential of non-pharmaceutical chemicals using primary human hepatocytes cultured in 3D spheroids. Spheroid cultures were repeatedly (co-) exposed to drugs (cyclosporine-A, bosentan, macitentan) or non-pharmaceutical chemicals (paraquat, tartrazine, triclosan) and a concentrated mixture of bile acids for 4 weeks. Cell viability (adenosine triphosphate content) was checked every week and used to calculate the cholestatic index, an indicator of cholestatic liability. Microarray analysis was performed at specific time-points to verify the deregulation of genes related to cholestasis, steatosis and fibrosis. Despite the evident inter-donor variability, shorter exposures to cyclosporine-A consistently produced cholestatic index values below 0.80 with transcriptomic data partially supporting its cholestatic burden. Bosentan confirmed to be hepatotoxic, while macitentan was not toxic in the tested concentrations. Prolonged exposure to paraquat suggested fibrotic potential, while triclosan markedly deregulated genes involved in different types of hepatotoxicity. These results support the applicability of primary human hepatocyte spheroids to study hepatotoxicity of non-pharmaceutical chemicals in vitro.

## 1. Introduction

Cholestasis denotes the disruption of bile synthesis or flow leading to the accumulation of toxic levels of bile acids (BAs) in hepatocytes or systemic circulation [1]. Cholestasis often results from the direct inhibition of hepatocellular canalicular transporters involved in the efflux of BAs, such as the bile salt export pump (BSEP, ABCB11) and multidrug resistance-associated protein (MRP) 2 (ABCC2) [2]. When triggered by drugs, cholestasis represents a type of drug-induced liver injury (DILI), which is a major clinical problem and one of the leading causes of drug withdrawal in premarketing and postmarketing phases of drug development [3]. This is due, at least in part, to interspecies differences between rodents and humans that result in poor predictability of DILI in preclinical animal models [4]. Furthermore, most human-based hepatic short-term in vitro models do not reflect the multiplicity and intricacy of the toxicity mechanisms involved in DILI, and therefore display limited predictability of the human situation [5]. For some patterns of DILI, such as cholestasis, the sometimes delayed onset of weeks to months after drug intake seems to be hardly mimicked in short-term in vitro settings [1].

There has been a great interest in overcoming the current limitations of preclinical studies by developing more reliable, in vivo-like in vitro models. Several years ago, a gene expression comparison demonstrated that adherent cryopreserved hepatocytes were equivalent to their freshly isolated counterparts and represent validated tools for drug metabolism and toxicological evaluations [6]. More recently, cryopreserved plateable human hepatocytes were kept as long-term culture, either as 2D-sandwich or as 3D-spheroids, thus making them a useful model for the assessment of repeated exposure-related toxicities [7,8,9]. Cultivation of PHH as 3D-spheroids, which uses a low cell number (1500 cells/3D spheroid), is compatible with a particularly high-throughput. Furthermore, the proteome of PHH spheroids closely resembles the human liver for over 7 days after full aggregation, and the molecular phenotypes and metabolic activity are largely stable for up to 5 weeks [8,9]. These 3D-spheroid cultures have been shown to form bile canaliculi [10] and to maintain drug metabolizing enzyme activities [11,12], which are of importance for drug toxicity events. The recently introduced drug-induced cholestasis assay in 2D-sandwich cultures, which makes use of the widely recognized mechanism of intracellular bile acid accumulation associated with cholestasis [13], could be successfully applied to PHH 3D-spheroid cultures [14], resulting in improved sensitivity of PHH towards the cholestatic toxicity of compounds under prolonged exposure. In these set-ups, PHH can be cultivated for up to a month, simulating scenarios of chronic, repeated, exposures to more realistic concentrations [8]. By concomitantly exposing the PHH spheroids to cholestatic drugs, such as cyclosporine-A (CyA) or bosentan (BOS), and a concentrated mixture of the most human-relevant BAs, the synergistic effects can be evaluated and a cholestatic index (CIx) can be calculated to translate such effect [13,15]. To contextualize the in vitro data, two safety margins (SM) in relation to the maximum drug concentration found in plasma from human volunteers (Cmax_total_) can be established, namely one that considers the highest drug concentration producing less than 20% drop in cell viability (SM_DILI_), and a second one taking the CIx (SM_CHOL_) into consideration [13,15]. A drug presenting an SM_DILI_ below 10 is considered to pose increased DILI risk [16], while an SM_CHOL_ below 30 indicates increased cholestatic risk [8]. Additionally, through the analysis of the transcriptomic changes induced by the chemical compounds, the compliance with the adverse outcome pathway (AOP) on drug-induced cholestasis can be investigated [17,18]. The AOP on drug-induced cholestasis describes the mechanistic basis of a drug-mediated cholestatic liver injury, starting with a drug-induced triggering factor or molecular initiating event, with a special focus on the inhibition of BSEP as main molecular initiating event [17]. Yet, hepatocellular changes, bile canalicular changes and other types of transporter disturbances are reported to play an at least as important role in inducing cholestasis [19]. These events typically evoke BAs accumulation, which in turn, can provoke two types of cellular responses, in particular the deteriorative response and the adaptive response. The former is characterized by inflammation, oxidative stress, mitochondrial stress, apoptosis and necrosis [17], and recently also endoplasmic reticulum (ER) stress, autophagy and necroptosis [18]. The adaptive response strives to counteract the deteriorative response, and thus cholestasis, by activating a number of nuclear receptors (NRs) that regulate genes involved in BA homeostasis [17,19]. In a recent study performed by our group, a genetic signature partially based on this AOP was also employed to depict non-pharmaceutical chemicals with assumed cholestatic characteristics but failed to identify genes relevant for cholestasis for all the treatments. This outcome could be attributed to a number of reasons, one of them being the need for a longer-term exposure screening method, which is not feasible in the human hepatoma HepaRG model, resulting in a relative lower sensitivity towards testing non-pharmaceutical compounds [20]. 

Besides the liver injury induced by drugs, a number of non-pharmaceutical chemicals have been reported to be a potential cause of hepatotoxicity, namely cholestasis, such as the herbicide paraquat (PQ), the food additive tartrazine (TAR) and the cosmetic ingredient triclosan (TRI), among others. PQ commercialization has been banned in the European Union in 2007 (Directive 91/414/EEC), but it is still responsible for many cases of involuntary poisoning all around the world [21]. A case of prolonged acute cholestasis has been reported after exposure to PQ absorbed via the skin [22]. The PQ serum levels in the patient were about 0.5 μg/mL (~2 μM) and the cholestatic pattern was still present 2 years after the diagnosis. The colorant TAR is used in cosmetics [23] and food [24], having been approved by the European Food Safety Agency with an acceptable daily intake of 7.5 mg/kg body weight/day [25]. It has been associated with cholestatic damage in rats [26] and in mice [27]. As an activator of the human estrogen receptor α [28], it may transcriptionally repress the expression of drug and BA transporters [29]. Our group recently identified high concentrations of TAR as potentially cholestatic in HepaRG cells [20]. TRI is an antimicrobial compound broadly used in products such as soaps, toothpaste and mouthwash, and it has been flagged as potentially cholestatic [23,30]. Its widespread presence in consumer products along with its rapid absorption via the skin and oral mucosa increase the likeliness of human exposure to TRI [31]. TRI was detected in 75% of the US population, reaching levels as high as 13 µM in urine [32]. Additionally, plasma levels after accidental ingestion of a mouthwash product containing 0.03% TRI approximated 1 μM [33] and steady-state levels have been estimated to reach 1.5–10 μM [34]. Still, in vitro mechanistic studies on the cholestatic effects of TRI are lacking. 

Considering the acknowledged applicability of the PHH spheroids for depicting DILI in a sensitive and human-relevant manner, this in vitro model was herein used, for the first time, to assess the hepatotoxic potential of non-pharmaceutical chemicals, which have been linked to the manifestation of cholestatic effects. For this purpose, spheroids from three different PHH donors were generated in the present study and used to investigate the ability of PQ, TAR and TRI to induce (cholestatic) hepatotoxicity. Hepatotoxic drugs, including CyA, BOS and macitentan (MAC), were used as benchmark chemicals. 

## 2. Results

### 2.1. Spheroid Generation

General characteristics of the PHH donors used for spheroid generation in this study are depicted in Table 1.

**Figure 1:**
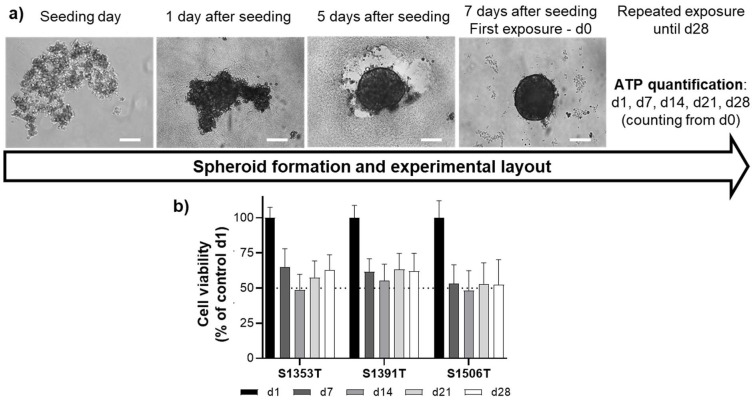
(**a**) Timeline of spheroid generation and experimental procedure. Cryopreserved PHH were seeded in ultra-low attachment plates at a density of 1500 cells per well. The plates were centrifuged (130× *g*, 2 min) and kept in controlled atmosphere (37 °C, 5% CO_2_, humidified). The spheroids were formed 5 days after seeding and the first exposure was performed 7 days after seeding (d0). Scale bar = 100 µm. (**b**) Cell viability of control spheroids throughout the time-frame of the experiments. Results are expressed as mean ± standard deviation of at least 48 spheroids from each donor per time-point. ATP—adenosine triphosphate; d—day.

For all donors, the PHH aggregated and formed compact spheroids within 5 days after seeding (Figure 1a). Independently of the PHH donor, spheroid viability was similarly high soon after the first exposure (day 1), dropping to about 50% on day 7, after which a stable viability was maintained towards day 28 (Figure 1b), which has been defined as an acceptable viability level [11]. This decline in cell viability is concurrent with a proportionate size reduction (Figure S1). When tested using the fluorescent probe 5(6)-carboxy-2′,7′-dichlorofluorescein diacetate (CDFDA), the 3D model exhibited functional bile canaliculi (Figure S2).

### 2.2. Concentration Selection and Concentration–Response Curves

For the concentration–response study, human-relevant concentrations of each test compound (Table 2) were selected following a literature search.

**Figure 2:**
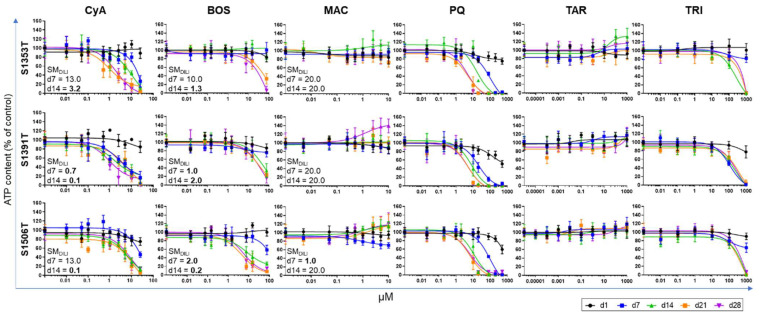
Cell viability of PHH spheroids after long-term repeated exposure to increasing concentrations of the test compounds. Concentration–response curves were obtained by quantifying total ATP content on days 1, 7, 14, 21 and 28 after first exposure. Concentrations were log-transformed and plotted using GraphPad Prism Software (Version 8.2.1). The DILI risk posed by the drugs was assessed by calculating the SM_DILI_ on days 7 and 14. Results are expressed as mean ± standard deviation of at least three spheroids per donor and per time-point. ATP—adenosine triphosphate; BOS—bosentan; CIx—cholestatic index; CyA—cyclosporine-A; d—day; MAC—macitentan; PQ—paraquat; SM_DILI_—safety margin for drug-induced liver injury; TAR—tartrazine; TRI—triclosan.

The evaluation of the individual responses to increased test compound concentrations provided a first hint of each donor’s susceptibility to the compounds, while defining the concentrations to be used in the subsequent experiments. The results from the concentration–response experiments showed that the three donors exhibit different sensitivity thresholds to most of the test compounds (Figure 2). For the drugs, the safety margin for hepatotoxicity (SM_DILI_) was estimated using Equation (1), considering the highest concentration producing less than 20% drop in adenosine triphosphate (ATP) content in proportion to the respective Cmax_total_. As for the non-pharmaceutical compounds, the unavailability of Cmax values in the literature hampers the respective SM_DILI_ calculation. An SM_DILI_ below 10 is suggestive of DILI risk [16].

Donor S1391T was in general more susceptible to the toxic effects of CyA and TRI compared to the other donors, while donor S1353T was more resistant to BOS, particularly at the earlier time-points. Despite these differences, the calculated SM_DILI_ for CyA and BOS was lower than 10 at day 14 for all donors, indicating potential human DILI risk. Such risk increased with time as on day 7 the SM_DILI_ remained above 10 for CyA in donors S1353T and S1506T (Figure 2). MAC did not produce a relevant toxic effect and the SM_DILI_ remained above 10. In fact, a tendency for increased ATP content was observed for longer exposure times to higher MAC concentrations. TAR caused a similar, though less prominent, increase in ATP content, while PQ steadily decreased ATP content, being cytotoxic to PHH from all donors for concentrations above 10 µM (Figure 2). Based on these results, in the subsequent study, CyA and BOS were tested at concentrations up to 10× the Cmax_total_, MAC up to 20× the Cmax_total_, PQ up to 10 µM, TAR up to 1000 µM and TRI up to 100 µM (Table 2).

### 2.3. Synergistic Effects between Test Compounds and Bile Acids

PHH spheroids were repeatedly exposed to selected concentrations of the test compounds (Table 2) in the presence or absence of a non-toxic 30× concentrated BAs mixture, and the ATP content was measured after 1, 7, 14, 21 and 28 days. The ratio between those two parameters was calculated (Table 3 and Table S1).

For the three donors, a CIx below 0.80, as defined in Oorts et al. [13], was consistently observed for CyA 5 µM, starting from day 7 (donors S1353T and S1506T) or day 14 (donor S1391T). This indicates that the added BAs potentiate in at least 20% the toxic effects of CyA. As for the interpretation of the DILI risk, the cholestatic risk was evaluated by calculating the SM_CHOL_, which related the lowest drug concentration yielding a CIx below 0.80 and the respective Cmax_total_ (Equation (3)). An SM_CHOL_ below 30 has been taken as indicative of cholestatic risk [14]. For CyA, the calculated SM_CHOL_ steadily remained under 30 (Table 4), which emphasizes the cholestatic potential of this drug. Curiously, BOS did not produce a consistent cholestatic pattern, and a definite CIx under 0.80 was only observed on day 21 or even later depending on the donor (Table S1). When analyzing the synergistic toxicity data (Figure S3), 75 µM BOS alone visibly decreased cell viability after prolonged exposure, preventing any robust conclusions on its cholestatic liability. Interestingly, at 7.5 µM BOS (i.e., Cmax_total_), the added BAs mix protected the spheroids from PHH donor S1506T from the toxic effect of BOS (CIx 2.34 ± 0.21 and 4.27 ± 0.28, Table S1, Figure S3), an effect previously described for acetaminophen [10]. As for MAC, donor S1391T yielded two positive results (i.e., CIx below 0.80, Table S1), but these were not confirmed at later time-points or higher concentrations. 

**Figure 3:**
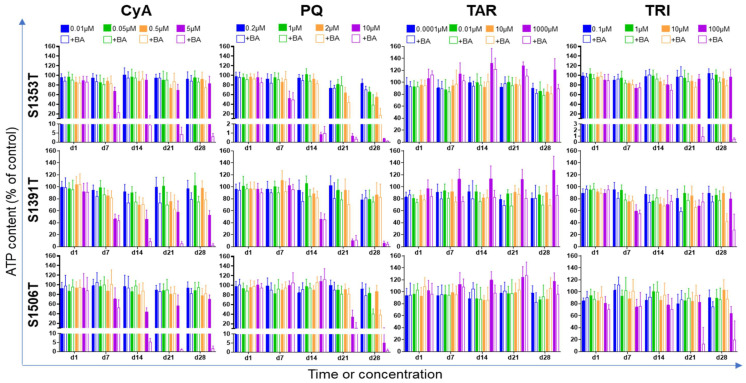
Effects of a concentrated BAs mixture on the hepatotoxicity of the test compounds throughout time. Spheroids from three different PHH donors were repeatedly incubated for 1, 7, 14, 21 and 28 days with the specified test compound in the presence (empty bars) and in the absence (filled bars) of a 30× concentrated mixture of BAs. At each predefined time-point, total ATP content was quantified. The effect of vehicle alone (control) was taken as 100%. Results are expressed as mean ± standard deviation of at least six spheroids per condition. ATP—adenosine triphosphate; BA—bile acid; CyA—cyclosporine-A; d—day; PQ—paraquat; TAR—tartrazine; TRI—triclosan.

PQ led to decreased CIx in donors S1353T and S1506T, particularly relevant at 1 and 2 µM, but not in donor S1391T. TAR similarly produced CIx under 0.80 in donors S1353T and S1391T, but a closer look at the synergistic toxicity data between TAR and BAs (Figure 3) suggests that these results might derive from an artefact introduced by the increased viability observed for prolonged exposure to 1000 µM TAR alone. In fact, when BAs were co-incubated with TAR 1000 µM, cell viability remained always above 80% comparatively to the untreated control. Unlike TAR, a very clear synergistic effect was observed between TRI 100 µM and the BAs mix after prolonged exposures (Table 3, Figure 3). The intensity of this effect was, however, more evident for donor S1353T (CIx 0.01 ± 0.01, Table 3) than for the other two donors.

Considering that the CIx below 0.80 for PQ 2 µM in two donors and for TRI 100 µM in the three donors indicated a potential cholestasic risk, the effects of these compounds at the mRNA level were further studied after a 28-day exposure, using CyA 5 µM as a reference for cholestatic effects. 

### 2.4. Transcriptomic Analysis

To investigate the hepatotoxic effects of non-pharmaceutical compounds PQ and TRI at gene level and evaluate whether a cholestatic genetic signature could be retrieved, a microarray analysis was performed on PHH spheroids that were repeatedly exposed to 2 µM PQ and 100 µM TRI for 28 days. PHH spheroids exposed to 5 µM CyA for 7 and 28 days were additionally included in the transcriptomic analysis as positive control for cholestasis [10,13]. An acute (7 days) and a chronic (28 days) setting were selected for the positive control to investigate the time element in retrieving cholestatic features at the transcript level and its compliance with the AOP on drug-induced cholestasis. 

**Figure 4:**
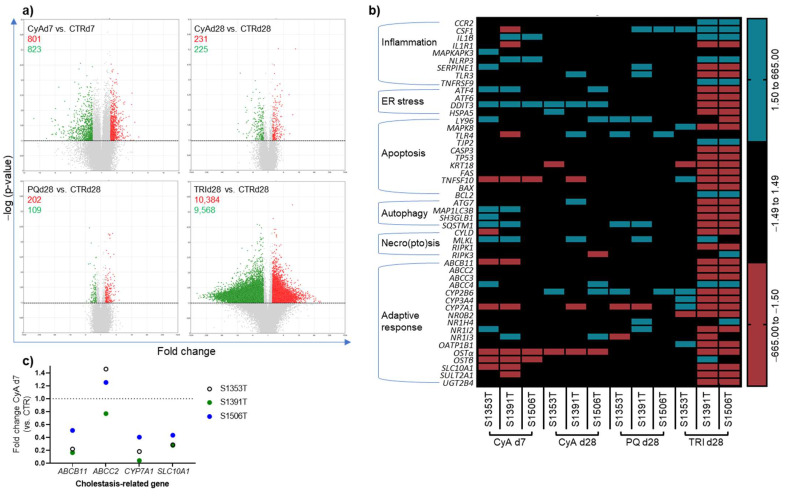
Transcriptomic analysis of PHH spheroids exposed to CyA 5 µM, PQ 2 µM and TRI 100 µM during 7 and/or 28 days. (**a**) Volcano plots displaying the differentially expressed genes between treated spheroids and the respective control, considering all donors. Significance was set at absolute fold changes above 1.5, with a *p*-value below 0.05 and an FDR *p*-value below 1, calculated via one-way ANOVA followed by post hoc tests using Bonferroni’s correction and Benjamini–Hochberg correction (green: downregulation; red: upregulation). (**b**) Heat-map displaying donor-by-donor compliance with the AOP on drug-induced cholestasis. At least 75 spheroids were used per donor and per condition. Absolute fold changes above 1.5 were considered significant. (**c**) RT-qPCR results confirming the effects of a 7-day exposure to CyA 5 µM on genes encoding canalicular transporters ABCB11 (BSEP) and ABCC2 (MRP2), uptake transporter SLC10A1 (NTCP), and CYP7A1, the main enzyme involved in BA synthesis and homeostasis. All the gene names are listed in Table S2 in the supplementary materials. ABC—ATP binding cassette; AOP—adverse outcome pathway; BSEP—bile salt export pump; CyA—cyclosporine-A; CYP—cytochrome P450; d—day; FDR—false discovery rate; MRP—multidrug resistance-associated protein; NTCP—sodium-taurocholate co-transporting polypeptide; PQ—paraquat, SLC—solute carrier; TRI triclosan.

According to the visual representation of the total number of significantly modulated genes (volcano plots, Figure 4a), a 3–4-fold higher number of differentially expressed genes was found after a 7-day (a total of 1624) comparatively to a 28-day (a total of 456) treatment with CyA. This highlights the importance of time-point selection for the transcriptomic analysis. On the other hand, prolonged PQ exposure resulted in the lowest number of significantly deregulated genes (total of 311), while TRI significantly modulated a striking total of 19,952 genes (Figure 4a). An analysis of the averaged global PHH response can, however, be deceiving, as it may hide an outlier response, as is the case of donor S1353T after TRI exposure (Figure 4a,b). Next, a more in-depth analysis was performed on specifically modulated genes relevant for cholestasis, using the updated AOP of cholestasis as benchmark [18]. All the genes tested in this study are summarized in Table S2 including their relevant biological role or function. In a glimpse, the transcriptomic responses to the tested compounds varied considerably between the three donors (Figure 4b). A 7-day exposure to CyA 5 µM resulted in upregulation of markers related to inflammation, such as interleukin *(IL)1β* and NOD-, LRR- and pyrin domain-containing protein 3 (*NLRP3*) in donors S1391T and S1506T, while donor S1353T showed an upregulation of MAP kinase-activated protein kinase 3 and serpin E1 (Figure 4b). The upregulation of activating transcription factor 4 in two donors, and of DNA damage-inducible transcript 3 in all donors supports an activation of the ER stress response. Additionally, genes related to autophagy, such as microtubule associated protein 1 light chain 3β and sequestosome, and relevant for necroptosis, as the mixed lineage kinase domain like pseudokinase (*MLKL*) gene, were found upregulated in two donors. Regarding the adaptive response described in the AOP on drug-induced cholestasis [17], only a few genes demonstrated to be in agreement with its activation. This is the case of the upregulation observed for ATP binding cassette *(ABC)C4* (or multidrug resistance-associated protein 4) and nuclear receptor *(NR)1I2* (or pregnane X receptor) in donor S1353T, and the downregulation of cytochrome P450 *(CYP)7A1* and solute carrier *(SLC)10A1* in donors S1353T and S1391T. In these two donors, *ABCB11* (or *BSEP*) has, in fact, been found downregulated by CyA. The results concerning *ABCB11*, *CYP7A1*, *ABCC2* and *SLC10A1* were confirmed by RT-qPCR analysis in PHH spheroids exposed during 7 days to CyA 5 µM (Figure 4c). Curiously, some of these changes, namely the ones concerning inflammation and autophagy, failed to be present after a 28-day exposure to CyA 5 µM (Figure 4b), again confirming the essentiality of time-point selection. With respect to a 28-day exposure to PQ 2 µM, only minor changes in the expression of genes related to drug-induced cholestasis could be observed in one or two donors, including a few genes related to inflammation (i.e., colony stimulating factor, serpin E1 and toll like receptor 3), apoptosis (i.e., lymphocyte antigen 96 and toll like receptor 4), autophagy (i.e., sequestosome 1) and necroptosis (i.e., *MLKL*) (Figure 4b). As for the adaptive response, only *CYP2B6* and *CYP7A1* were simultaneously affected by PQ in two donors. TRI, on the other hand, seemed to induce a rather unstable genetic phenotype (Figure 4b). Indeed, while many inflammation markers were upregulated (e.g., C-C motif chemokine receptor 2, colony stimulating factor 1, *IL1β* and *NLRP3*), others were downregulated (e.g., IL1 receptor 1, *SERPINE1* and toll like receptor 3). Yet, overall a particular downward trend could be distinguished with the majority of differentially regulated genes being downregulated, such as cholestasis mechanisms ER stress, apoptosis, autophagy and adaptive response-related markers (Figure 4b). In this regard, the downregulation of *CYP7A1*, organic anion transporter 1B1 and *SLC10A1* in two donors is compliant with the AOP on drug-induced cholestasis [17] and confirms results previously obtained in spheroid cultures of HepaRG cells and in PHH in long-term 2D-sandwich culture [18,20]. Concerning donor S1353T, only a few genes related to cholestasis were upregulated by TRI 100 µM (Figure 4b). Of interest are the genes encoding CYP2B6, CYP3A4 and CYP7A1, from which the former is implicated in TRI metabolism, while *CYP3A4* and *CYP7A1* are involved in BA synthesis, hence contributing for BA accumulation in hepatocytes.

**Figure 5:**
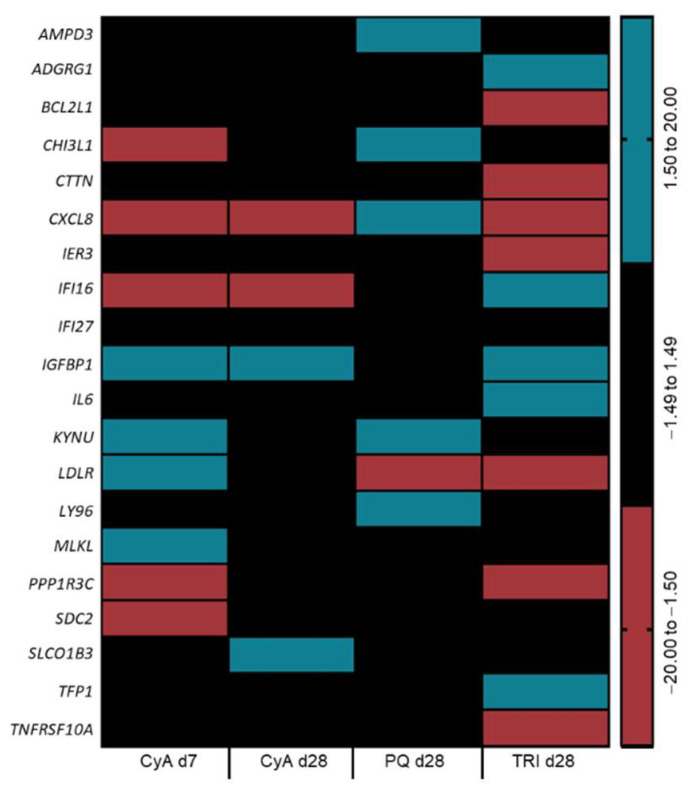
Identification of biomarkers of drug-induced cholestasis according to [20], per condition. All the gene names are listed in Table S2 in the supplementary materials. Averaged results from spheroids from three PHH donors. CyA—cyclosporine-A; d—day; PHH—primary human hepatocytes; PQ—paraquat; TRI—triclosan.

Similar to the AOP strategy, transcriptomic data obtained from the tested compounds were benchmarked to a list of genetic biomarkers of chemical-induced cholestasis established by our group [18,20] (Figure 5).

A 7-day exposure of the PHH spheroid model to CyA 5 µM resulted in enhanced compliance with the cholestasis biomarkers compared to a 28-day exposure, evidenced by a downregulation of chitinase 3 like 1, protein phosphatase 1 regulatory 3C (*PPP1R-3C*) and syndecan 2, and an upregulation of insulin like growth factor binding protein 1 (*IGFBP1*), kynureninase, low density lipoprotein receptor and *MLKL* (Figure 5). Interestingly, PHH spheroids exposed to TRI 100 µM for 28 days also displayed six deregulated biomarker genes in agreement with previous studies, namely adhesion G protein-coupled receptor G1, interferon ɣ inducible protein 16, *IGFBP1*, *IL6*, *PPP1R-3C* and tissue factor pathway inhibitor (Figure 5), therefore reinforcing the suspicion on the cholestatic potential of TRI.

**Figure 6:**
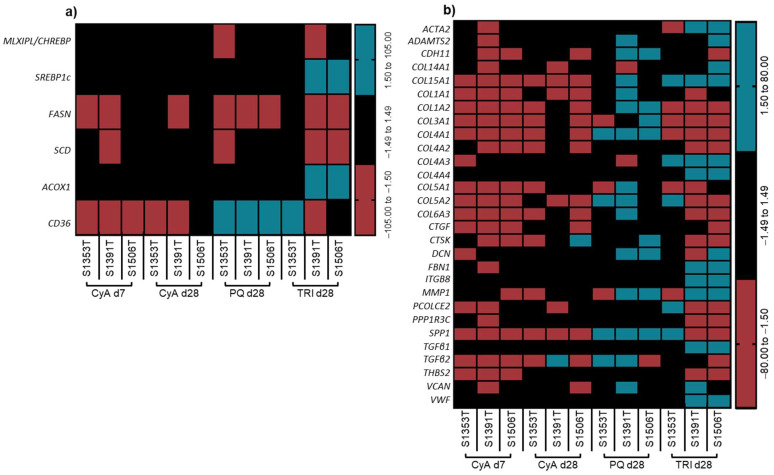
Relative expression of genes related to (**a**) liver steatosis and (**b**) liver fibrosis. The effects of a 7- and 28-day exposure to CyA 5 µM and of a 28-day exposure to PQ 2 µM and TRI 100 µM were assessed by microarray analysis in at least 75 spheroids from each PHH donor. Absolute fold changes in gene expression above 1.5 were considered significant. All the gene names are listed in Table S2 in the supplementary materials. CyA—cyclosporine-A; d—day; PHH—primary human hepatocytes; PQ—paraquat; TRI—triclosan.

Based on the discrepancies between the obtained transcriptomic signature and the cholestatic signature described in our previous works [18,20], no unambiguous prediction could be made on the cholestatic potential of the non-pharmaceutical compounds TRI and PQ, raising the question whether other hepatotoxic effects could be involved. Therefore, the obtained transcriptomic signature of the tested compounds in PHH spheroids was additionally compared to gene lists entangled in other common types of DILI, namely steatosis and fibrosis [39,40] (Figure 6). CyA 5 µM appeared not to induce steatosis or fibrosis in PHH spheroids after 7 or 28 days of exposure, as the majority of modulated genes appeared downregulated in contrast to the predicted activity in both liver pathologies. In contrast to CyA, PQ 2 µM induced the expression of cluster determinant 36, a fatty acid translocase, which might indicate a dysfunction in the fatty acid metabolism (Figure 6a). More importantly, PQ also induced a number of genes involved in liver fibrosis (Figure 6b) in either two or three donors, including α chains of collagen (*COL*), cadherin 11, decorin, transforming tumor growth factor *(TGF)β2* and secreted phosphoprotein 1.

Similar to its effect on cholestasis-related genes, TRI caused a general downregulation of genes involved in steatotic and fibrotic processes in donors S1391T and S1506T (Figure 6a,b). Interestingly, however, all donors experienced an upregulation in COL type XV α1 (*COL15A1*) and *COL4A3*, and two donors additionally exhibited an upregulation of actin α2, *COL4A4*, fibrilin 1, integrin β8, *TGFβ1* and Von Willebrand factor (Figure 6b). The deregulation of six fibrosis-related genes, including the downregulation of matrix metallopeptidase 1, in spheroids from PHH donor S1353T suggests some degree of fibrotic liability for TRI (Figure 6b).

## 3. Discussion

A plethora of in vitro models are currently available presumed fit for modeling and predicting DILI, including drug-induced cholestasis [13,19,41,42,43]. However, only a limited number of models are capable of testing in a long-term, repeated exposure setting, allowing a more sensitive approach to testing compounds [19]. For this reason, PHH spheroids are considered to have high potential for screening hepatotoxic drugs [5,8,10,35,43,44]. This study aimed to expand their applicability domain and evaluate their ability to depict the (cholestatic) hepatotoxic potential induced by non-pharmaceutical chemicals. Accordingly, drugs with identified cholestatic liability (i.e., CyA, BOS and MAC) and non-pharmaceutical chemicals (i.e., PQ, TAR and TRI) with suspected, though less clear, cholestatic potential were tested in spheroids generated from PHH from three distinct donors. PHH spheroids were exposed to the test compounds for different periods of time, in the presence or absence of a 30× concentrated BAs mixture, which has been described as the highest non-toxic concentration in long-term cultures [14]. The initial check on the cholestatic liability of each test compound was based on their CIx being equal to or lower than 0.80, i.e., when the co-incubation with the concentrated BAs mixture resulted in at least 20% reduction of the total ATP content comparatively to the test compound alone [10,13,18,20].

The concentrations used in this study were based on reported Cmax_total_ values of the tested drugs [35,36,37], to be representative of the human scenario. As expected, CyA and BOS produced a concentration- and time-dependent decrease in cell viability. MAC, on the other hand, led to an interesting protective effect at higher concentrations or longer time-points, as supported by the increased cell viability. When co-exposed to the BAs mixture, an evident synergistic effect was observed for CyA, consistently yielding a CIx below 0.80, indicative of cholestatic liability, from day 7 and day 14 onwards. These results are in line with previous reports on CIx values at day 7 (i.e., 0.22) and day 14 (i.e., 0.01) using 20 µM CyA [14]. In particular, donor S1391T was more susceptible than the remaining to CyA 5 µM, as denoted by the SM_DILI_ far below 10 on day 7, together with the decreasing SM_CHOL_ and the delayed drop in the CIx at concentrations in the infra-therapeutic range described for low dose treatments (0.08–0.20 µM [45]). These results confirm those previously observed in PHH from this donor in 2D sandwich culture, depicting cholestatic potential of 20 µM CyA after 48 h of exposure [14]. Contrarily to CyA, BOS did not produce consistent cholestatic pattern when co-incubated with the BAs mix. For donors S1353T and S1506T, the average CIx ultimately fell below 0.80 using higher concentrations, yet with high standard deviations and with increased toxicity observed for BOS alone at 75 µM. These results follow previous reports where no clear cholestatic effect was observed for BOS in sandwich cultured human hepatocytes [13,15]. Regardless of the unclear cholestatic liability of BOS, the calculated SM_DILI_ below 10 indicates that all the donors face potential risk of DILI after prolonged treatment with BOS. As for MAC, this is the first time, to our knowledge, that this drug was tested in the PHH spheroid model. Even though MAC has been described to induce cholestatic features in HepaRG cells [46], this study failed to reproduce those findings. Patients treated with MAC also did not experience increased hepatic toxicity comparatively to those taking placebo [47]. Therefore, according to the data from this study, MAC presents low cholestatic DILI risk in the tested concentrations.

For the non-pharmaceutical compounds, the selected concentration ranges include serum levels, estimated human exposure levels, or steady-state concentrations described in literature reports [22,28,31,32,33,34,38,48]. Interestingly, the present study could recapitulate the previously reported delayed cholestatic liability of 2 µM PQ in the spheroids from PHH donors S1353T and S1506T, as denoted by the CIx below 0.80, unlike our previous study in HepaRG cells [20]. This is supportive of the higher sensitivity of the PHH spheroid model. In this study, TAR was generally well tolerated, even preserving cell viability, or promoting cell proliferation, at higher concentrations, as previously observed [38]. Even though a CIx under 0.80 was calculated for TAR 1 mM, this might relate to the mentioned preserving effect because co-incubation with BAs did not decrease the viability of the spheroids below that of the control. Considering that human exposure after consumption within the acceptable daily intake of TAR has been estimated between 1 pM and 10 nM [38], and that a clear-cut synergistic effect of the BAs was only observed for at least 1000× higher concentrations (10 µM or more), TAR seems not to represent an immediate cholestatic risk. In this study, a very low CIx was consistently observed at the end of the experiment for 100 µM TRI, underscoring its cholestatic liability.

As the CIx method is merely based on one endpoint, this study additionally integrated transcriptomic analysis for the validation of cholestatic DILI in PHH spheroids exposed to CyA 5 µM, TRI and PQ. In line with earlier studies on PHH-based models, CyA was taken as a positive control for drug-induced cholestasis [7,10,13,14], emulating a more acute exposure (7 days) and a chronic exposure (28 days), to scrutinize the time component in drug-induced cholestasis in PHH spheroids. In this regard, a 7-day exposure to CyA resulted in upregulation of genes related to inflammation, autophagy and ER stress, which has been described as a core element in CyA-induced cholestasis [49]. The activation of the adaptive response was also visible with an upregulation of either *NR1I3* or *NR1I2* together with the downregulation of *CYP7A1* and *SLC10A1*, which could be considered an attempt to counteract the BA accumulation derived from the concomitant *ABCB11* downregulation, known as a trigger of cholestasis [17]. A similar trend has been observed in HepaRG cells exposed for 24 h to 50 µM CyA [50]. Curiously, upon prolonged exposure to CyA, the deregulation in inflammation-related and autophagy-related genes appeared to be lost, demonstrating the importance of the selection of an appropriate time-point for the transcriptomic analysis. On the other hand, a continuous upregulation of the ER stress genes could be observed on both days 7 and 28 reinforcing the role of CyA in ER stress induction [49]. In addition, these findings also emphasize the robustness of ER stress as key event in cholestasis, in multiple in vitro models, including PHH spheroids (this study) and monolayer HepaRG cells, different presumed cholestatic compounds (i.e., CyA, atazanavir, nefazodone and TAR) and across time (i.e., acute and chronic setting) [18,20].

As for TRI and PQ, transcriptomic data did not align with the findings from the CIx method, as a minimal number of genes appeared modulated in compliance with the AOP on cholestasis. However, the transcriptomic profile of two donors (S1391T and S1506T, males) being exposed to TRI clearly suggested disrupted hepatocyte homeostasis. Even though we cannot exclude the existence of gender-related differences in the response to TRI, the relative decreased sensitivity of donor S1353T (female) towards TRI alone might be explained by the observed upregulation of *CYP2B6*, as previously denoted [51]. Likewise, the increased susceptibility to the co-incubation with BAs could be derived from the observed upregulation of *CYP7A1* possibly leading to increased basal BA levels.

Since a cholestatic transcriptomic profile could not be validated in PHH spheroids exposed to non-pharmaceutical compounds TRI and PQ, other types of hepatotoxicity, such as steatosis and fibrosis, were investigated in their obtained transcriptomic profile. Of note, the selected positive control for cholestasis, CyA, has also been reported to induce steatotic features in PHH spheroids [8] and in HepaRG cells [52]. However, the results from the present study cannot support those findings. Similarly, modulated genes resultant from TRI exposure were not in compliance with the predicted transcriptomic profile of steatosis nor fibrosis. In fact, TRI might not evoke steatosis on its own, rather requiring a co-exposure with fatty acids, as observed in a recent animal study in which TRI induced changes in all aspects of energy balance [53]. Only one study is available describing fibrosis after TRI exposure, yet this study used unrealistically high levels of TRI (i.e., 15–68.6 mg/kg), not relevant for human exposure (i.e., ∼0.05 mg/kg) [54]. Interestingly, PQ showed some compliance with a fibrotic response characterized by the upregulation of many collagen type genes, *TGFβ2* and osteopontin, as has been evidenced in rats treated with PQ [55].

Importantly, upon considering the PHH spheroids as model for prediction of (cholestatic) hepatotoxicity, inter-donor differences must be kept in mind. Indeed, the three donors responded quite distinctively to the test compounds and to the co-incubation with the 30× concentrated BAs mixture, except for MAC and TAR, which were mostly non-toxic. This supports the preservation of inter-donor variability in vitro, as previously demonstrated [7,13,14].

In conclusion, this work represents an exploratory effort to expand the use of the PHH spheroid model to study (cholestatic) hepatotoxic features induced by non-pharmaceutical chemicals. While TAR, BOS and MAC were considered to pose low cholestatic risk to the tested donors, the data support fibrosis-inducing potential for PQ, while TRI clearly distressed hepatocyte homeostasis. The results herein reported also highlight the importance of choosing an adequate time-point to evaluate transcriptomic changes and support the maintenance of variability in individual responses. Some of the identified limitations of this work include the low number of studied donors and tested chemicals, and the need to confirm the functionality of the spheroids from all PHH donors. Therefore, follow-up studies are necessary to confirm this data and to overcome the limitations of this work, paying special attention to gender representation in order to avoid gender bias, and to the thorough characterization of model functionality.

## 4. Materials and Methods

### 4.1. Reagents and Chemicals

Universal cryopreservation recovery medium (81015) was purchased from In Vitro ADMET Laboratories, Inc. (Columbia, MD, USA). Fetal bovine serum Hyclone was purchased from GE Healthcare (Diegem, Belgium). William’s E medium (no phenol red, Gibco A12176) and insulin-transferrin-selenium (41400045) were provided by ThermoFisher Scientific (Merelbeke, Belgium). L-glutamine-penicillin-streptomycin solution (G1146), dexamethasone (D1756), glycochenodeoxycholic acid (G0759), deoxycholic acid (30970), chenodeoxycholic acid (C9377), glycocholic acid (G2878), glycodeoxycholic acid (361311), dimethyl sulfoxide (472301), paraquat (PQ, 36541), tartrazine (TAR, T0388), triclosan (TRI, 72779) were purchased from Sigma-Aldrich (Overijse, Belgium). Cyclosporine-A (CyA, 239835) was purchased from Calbiochem (Darmstadt, Germany).

### 4.2. Primary Human Hepatocytes Seeding and Spheroid Generation

All the experiments were performed using cryopreserved PHH (KaLy-Cell, Plobsheim, France) from 3 distinct donors (Table 1). PHH were seeded by following a previously established protocol [8]. Briefly, PHH were thawed in universal cryopreservation recovery medium at 37 °C and centrifuged for 10 min at 100× *g*. After decanting the thawing medium, the pellet was dispersed in seeding cell culture medium (William’s E medium supplemented with 2 mM L-glutamine, 100 units/mL penicillin, 100 μg/mL streptomycin, 10 μg/mL insulin, 5.5 μg/mL transferrin, 6.7 ng/mL sodium selenite, 100 nM dexamethasone and 10% fetal bovine serum). Cell viability was estimated by counting viable and non-viable cells with Trypan Blue (Bio-Rad, Temse, Belgium). For spheroid generation, 1500 cells per well were seeded in 96-well ultra-low attachment plates (Corning, Amsterdam, Netherlands). The plates were gently centrifuged at 130× *g* for 2 min and then kept in an incubator with controlled atmosphere (37 °C, 5% CO_2_). Five days after seeding, spheroids were formed and half of the cell culture medium was exchanged for serum-free cell culture medium, further referred to as PHH medium. The exposure of the PHH spheroids to drugs and non-pharmaceutical chemicals was initiated 7 days after seeding and repeated every 2–3 days until 28 days after first exposure, by gently aspirating the cell culture medium and dispensing freshly prepared solutions.

### 4.3. Adenosine Triphosphate Quantification

The effects on cell viability were evaluated after 1, 7, 14, 21 and 28 days by measuring total adenosine triphosphate (ATP) content. To concentrate the bioluminescence signal, 70 μL of PHH medium was removed per well and 30 μL of Cell TiterGlo 3D reagent (G9682, Promega, Leiden, Netherlands) was added. The PHH spheroids were disrupted by vigorously pipetting up and down and incubating the plate for 20 min at 37 °C in the dark. Subsequently, 50 μL per well were transferred to a white plate (Lumitrac, Greiner Bio-One GmbH, Frickenhausen, Germany) and bioluminescence signal was acquired using a Victor3^™^ Multilabel Plate Reader (PerkinElmer, Zaventem, Belgium).

### 4.4. Concentration–Response Curves

Stock solutions were prepared in DMSO except for PQ and TAR, which were prepared in ultra-pure water (Milli-Q^®^, Merck Millipore, Darmstadt. Germany). From these, growing concentrations of drugs or non-pharmaceutical chemicals were prepared in PHH medium according to Table 2. On days 1, 7, 14, 21 and 28 after first exposure, the effects on cell viability were evaluated by quantifying total ATP content. A concentration–response curve was independently obtained for each compound at 5 different time-points, for each donor. With these results, the SM_DILI_ was calculated using the following equation:

(1) $${\mathrm{SM}}_{\mathrm{DILI}}=\frac{\mathrm{highest}\;\mathrm{drug}\;\mathrm{concentration}\;\mathrm{yielding}>80\%\;\mathrm{ATP}\;\mathrm{content}}{{\mathrm{Cmax}}_{\mathrm{total}}}$$

### 4.5. Evaluation of Synergistic Effects and Cholestatic Index Calculation

The CIx is calculated by evaluating the synergistic effects of a concentrated BA mixture on the cytotoxicity induced by a test compound [10]. PHH spheroids were repeatedly exposed to test compounds, in the presence or in the absence of a 30× concentrated BA mix. The 30× concentrated BA mix was composed of the 5 most relevant bile acids for humans, namely 39.6 μM glycochenodeoxycholic acid, 11.7 μM chenodeoxycholic acid, 11.4 μM glycodeoxycholic acid, 12 μM deoxycholic acid and 10.5 μM glycocholic acid [15]. Test compound concentrations were selected based on the overall data obtained from the concentration–response curves (Table 2). On days 1, 7, 14, 21 and 28 after first exposure, the effects on cell viability were evaluated by quantifying total ATP content and the CIx was calculated using the following equation:

(2) $$\mathrm{CIx}=\frac{\mathrm{ATP}\;\mathrm{content}\;\left(\mathrm{compound}+\mathrm{BA}\;\mathrm{mix}\right)}{\mathrm{ATP}\;\mathrm{content}\;\mathrm{compound}\;\mathrm{alone}}$$

With the results of the CIx, the SM_CHOL_ was calculated using Equation (3):

(3) $${\mathrm{SM}}_{\mathrm{CHOL}}=\frac{\mathrm{lowest}\;\mathrm{drug}\;\mathrm{concentration}\;\mathrm{yielding}\;\mathrm{CIx}<0.80}{{\mathrm{Cmax}}_{\mathrm{total}}}$$

### 4.6. RNA Extraction

RNA was extracted from approximately 90 PHH spheroids per condition (CTR, CyA 5 μM, PQ 2 μM and TRI 100 μM) and per donor, after a 7-day and/or 28-day repeated exposure period, using an RNeasy Mini kit (Qiagen, Hilden, Germany). Quantification and purity of the extracted RNA were evaluated by means of spectrophotometric analysis with a Nanodrop spectrophotometer (ThermoFisher Scientific, Merelbeke, Belgium).

#### 4.6.1. Reverse Transcription Quantitative Polymerase Chain Reaction

In total, 150 ng of mRNA were reverse-transcribed (iScript cDNA synthesis kit, Bio-Rad, Temse, Belgium) into cDNA and purified (GenElute™ PCR Clean-Up Kit, Sigma-Aldrich, Overijse, Belgium). For the reverse transcription quantitative polymerase chain reaction (RT-qPCR) analysis, 10 ng of cDNA were combined with Taqman^®^ Fast Advanced Mastermix (Applied Biosystems, Waltham, MA, USA) and Taqman primers (ABCB11, Hs00184824_m1; ABCC2, Hs00960489_m1; CYP7A1, Hs00167982_m1; SLC10A1, Hs00161820_m1; and TBP, Hs00427620_m1), and amplified in a StepOnePlus™ real-time PCR system (Applied Biosystems, Waltham, MA, USA). Fold changes in the RNA levels were calculated and corrected for the PCR efficiency of each gene using Equation (4) [56]:

(4) $$\mathrm{ratio}=\frac{{\left(E_{\mathrm{target}}\right)}^{\Delta \mathrm{CPtarget}\;\left(\mathrm{control}\;-\;\mathrm{sample}\right)}}{{\left(E_{\mathrm{ref}}\right)}^{\Delta \mathrm{CPref}\;\left(\mathrm{control}\;-\;\mathrm{sample}\right)}}$$

in which E_target_ is the PCR efficiency of the target gene and E_ref_ that of the housekeeping gene, ΔCP_target_ is the difference between the crossing points (CP) of the control and test sample for the target gene, and ΔCP_ref_ is the same difference but for the housekeeping gene. PCR efficiency was calculated (E = 10^(−1/slope)^) considering the slope of the standard curve obtained when plotting the log10 of the cDNA dilution and the respective CP for each tested gene.

#### 4.6.2. Microarray Analysis and Transcriptomic Data Processing

Whole genome expression analysis was performed using microarray technologies from Affymetrix (Santa Clara, CA, USA) as previously described [18,57]. For this purpose, biotinylated ds-cDNA was prepared from 2 ng total RNA using GeneChip™ 3′ IVT Pico Kit (Thermo Fisher Scientific, Merelbeke, Belgium) and then hybridized onto GeneChip™ Human Genome U133 Plus 2.0 Assay chips (Thermo Fisher Scientific, Belgium). Subsequently, the chips were placed in a GeneChip™ Hybridization Oven 645 (Affymetrix, Santa Clara, CA, USA) following manufacturer’s instructions. After incubation, the arrays were washed with GeneChip™ Fluidics Station 450 (Affymetrix, Santa Clara, CA, USA) and stained with Affymetrix HWS kit. Thereafter, stained arrays were scanned using an Affymetrix GeneChip™ Scanner 3000 7G (Affymetrix, Santa Clara, CA, USA). Hybridization controls were performed using Affymetrix GeneChip™ Operating Software. Normalization quality controls, such as scaling factors, background intensities, noise and raw Q-values, average intensities and present calls were done with RMA Express software and were all within the acceptable limits. Volcano plots were performed using Transcriptome Analysis Console (version 4.0.025, Affymetrix, Santa Clara, CA, USA) software, with significance set when an absolute fold change exceeded 1.5 with a *p*-value below 0.05 and an FDR *p*-value below 0.05. Heat-maps summarizing deregulated genes (significance set at fold change threshold of ±1.5 [7]) for each donor were produced using GraphPad Prism software.

## Figures and Tables

**Table 1:** Demographics of the donors of the cryopreserved PHH used in this study.

Donor	Gender	Age	Pathology
S1353T	Male	62	Sigmoid adenocarcinoma
S1391T	Female	54	Non-transplantable liver
S1506T	Female	47	Adenocarcinoma

**Table 2:** Concentrations of test compounds for the concentration–response curve and the CIx experiments.

Test Compound	Tested Concentration (µM)		Human-Relevant Concentration (µM)
	Concentration-Response	CIx	
BOS	0; 0.015; 0.075; 0.15; 0.75; 1.5; 7.5; 15; 75	0; 0.15; 1.5; 7.5; 75	7.5 *
CyA	0; 0.01; 0.05; 0.1; 0.5; 1; 2.5; 5; 10; 25	0; 0.01; 0.05; 0.5; 5	0.8 *
MAC	0; 0.005; 0.05; 0.25; 0.5; 1; 2.5; 5; 10	0; 0.005; 0.5; 5; 10	0.5 *
PQ	0; 0.02; 0.2; 1; 2; 10; 20; 80; 200; 500	0; 0.2; 1; 2; 10	2.0 ^#^
TAR	0; 0.0001; 0.001; 0.01; 0.1; 1; 10; 100; 1000	0; 0.0001; 0.01; 10; 1000	0.01 ^$^
TRI	0; 0.01; 0.1; 1; 10; 100; 1000	0; 0.1; 1; 10; 100	10 ^&^

* Cmax_total_ [35,36,37], ^#^ serum level in patient with cholestasis [22], ^$^ estimated human exposure [38], ^&^ maximum estimated steady-state concentration [34]. BOS—bosentan; CIx—cholestatic index; CyA—cyclosporine-A; MAC—macitentan; PQ—paraquat; TAR—tartrazine; TRI—triclosan.

**Table 3:** Inter-donor variability regarding the effect of drug/chemical concentration and time of exposure on the cholestatic index values.

		CyA				PQ				TAR				TRI			
	**µM**	**0.01**	**0.05**	**0.5**	**5**	**0.2**	**1**	**2**	**10**	**0.0001**	**0.01**	**10**	**1000**	**0.1**	**1**	**10**	**100**
**S1353T**	d1	0.92 ± 0.00	0.93 ± 0.04	0.96 ± 0.16	0.97 ± 0.08	0.98 ± 0.06	0.95 ± 0.08	0.96 ± 0.11	0.88 ± 0.00	0.97 ± 0.07	1.00 ± 0.02	1.01 ± 0.16	1.06 ± 0.09	0.98 ± 0.02	0.91 ± 0.02	1.02 ± 0.09	0.99 ± 0.05
	d7	0.92 ± 0.06	0.94 ± 0.07	0.94 ± 0.18	0.33 ± 0.18	0.90 ± 0.10	0.98 ± 0.09	1.06 ± 0.18	0.94 ± 0.08	0.98 ± 0.00	0.98 ± 0.13	1.04 ± 0.20	0.90 ± 0.05	1.02 ± 0.05	0.91 ± 0.13	0.97 ± 0.00	1.01 ± 0.08
	d14	0.94 ± 0.07	0.96 ± 0.16	1.03 ± 0.11	0.11 ± 0.04	0.94 ± 0.01	0.94 ± 0.01	0.90 ± 0.01	1.40 ± 0.08	0.93 ± 0.11	1.01 ± 0.00	1.08 ± 0.19	0.90 ± 0.00	1.05 ± 0.11	0.94 ± 0.14	0.93 ± 0.00	0.82 ± 0.07
	d21	1.00 ± 0.04	0.99 ± 0.00	1.06 ± 0.07	0.06 ± 0.07	1.00 ± 0.20	0.94 ± 0.09	0.68 ± 0.07	0.54 ± 0.16	1.06 ± 0.00	0.94 ± 0.12	0.98 ± 0.04	0.87 ± 0.01	0.98 ± 0.07	0.91 ± 0.00	0.87 ± 0.12	0.01 ± 0.01
	d28	1.00 ± 0.23	0.91 ± 0.13	0.82 ± 0.01	0.04 ± 0.02	0.84 ± 0.07	0.52 ± 0.30	0.32 ± 0.08	0.28 ± 0.04	0.89 ± 0.07	0.95 ± 0.15	0.96 ± 0.05	0.74 ± 0.10	0.89 ± 0.05	0.84 ± 0.05	0.89 ± 0.11	0.01 ± 0.00
**S1391T**	d1	1.03 ± 0.14	0.98 ± 0.09	1.01 ± 0.09	0.98 ± 0.07	1.00 ± 0.12	0.93 ± 0.14	0.99 ± 0.01	0.92 ± 0.09	1.03 ± 0.08	0.92 ± 0.05	0.92 ± 0.01	0.88 ± 0.18	1.05 ± 0.12	1.02 ± 0.05	0.97 ± 0.09	1.04 ± 0.09
	d7	0.90 ± 0.02	0.88 ± 0.02	0.95 ± 0.09	0.93 ± 0.08	0.93 ± 0.13	0.90 ± 0.12	0.84 ±0.04	0.93 ± 0.07	0.98 ± 0.12	0.94 ± 0.08	0.90 ± 0.05	0.73 ± 0.07	0.85 ± 0.02	0.89 ± 0.11	1.01 ± 0.14	0.85 ± 0.04
	d14	0.81 ± 0.09	0.83 ± 0.12	1.01 ± 0.06	0.21 ± 0.13	0.82 ± 0.02	0.80 ± 0.02	0.94 ± 0.16	1.05 ± 0.15	0.87 ± 0.00	0.82 ± 0.01	0.95 ± 0.08	0.77 ± 0.02	0.87 ± 0.03	1.08 ± 0.27	0.96 ± 0.14	1.11 ± 0.32
	d21	0.80 ± 0.02	0.83 ± 0.21	0.77 ± 0.18	0.09 ± 0.07	0.83 ± 0.04	0.85 ± 0.00	0.91 ± 0.08	1.08 ± 0.05	0.87 ± 0.10	0.83 ± 0.02	0.97 ± 0.05	0.73 ± 0.16	0.79 ± 0.02	0.90 ± 0.13	0.80 ± 0.01	1.10 ± 0.16
	d28	0.83 ± 0.02	0.79 ± 0.03	0.73 ± 0.16	0.08 ± 0.04	1.06 ± 0.16	0.93 ± 0.17	0.94 ± 0.13	1.07 ± 0.16	1.01 ± 0.07	0.80 ± 0.14	0.75 ± 0.04	0.67 ± 0.13	0.84 ± 0.00	0.90 ± 0.04	0.48 ± 0.05	0.54 ± 0.13
**S1506T**	d1	0.98 ± 0.22	1.10 ± 0.03	1.01 ± 0.02	1.03 ± 0.13	1.01 ± 0.12	0.95 ± 0.04	1.01 ± 0.22	0.92 ± 0.04	1.01 ± 0.14	1.06 ± 0.02	1.18 ± 0.11	0.95 ± 0.06	1.05 ± 0.11	0.93 ± 0.10	1.11 ± 0.12	0.87 ± 0.07
	d7	1.07 ± 0.03	1.06 ± 0.13	1.09 ± 0.15	0.77 ± 0.08	0.91 ± 0.07	1.11 ± 0.01	1.06 ± 0.02	1.05 ± 0.15	1.01 ± 0.12	1.00 ± 0.04	0.98 ± 0.05	0.98 ± 0.25	1.08 ± 0.15	1.09 ± 0.16	1.12 ± 0.09	1.04 ± 0.25
	d14	0.88 ± 0.01	1.13 ± 0.00	1.01 ± 0.20	0.12 ± 0.05	0.99 ± 0.13	0.99 ± 0.08	1.18 ± 0.01	1.03 ± 0.00	1.18 ± 0.04	1.05 ± 0.26	1.03 ± 0.19	0.83 ± 0.06	1.00 ± 0.26	0.96 ± 0.14	1.12 ± 0.07	0.90 ± 0.11
	d21	0.94 ± 0.15	1.02 ± 0.13	1.01 ± 0.20	0.02 ± 0.01	0.91 ± 0.13	0.96 ± 0.18	0.93 ± 0.07	0.20 ± 0.04	1.05 ± 0.07	1.02 ± 0.08	1.04 ± 0.15	1.03 ± 0.15	1.08 ± 0.22	1.06 ± 0.08	1.05 ± 0.02	0.27 ± 0.47
	d28	0.88 ± 0.06	1.06 ± 0.28	1.04 ± 0.02	0.02 ± 0.02	0.87 ± 0.07	0.45 ± 0.10	0.45 ± 0.04	0.22 ± 0.13	0.87 ± 0.06	1.00 ± 0.21	1.25 ± 0.12	0.87 ± 0.15	0.86 ± 0.06	0.96 ± 0.23	0.84 ± 0.35	0.35 ± 0.60

Values were calculated for each of the test compounds in four different concentrations in the presence and absence of concentrated BAs mixture, at five consecutive time-points, for each of the three donors. Data represent mean ± standard deviation values collected from at least six spheroids per condition. CIx values below 0.80 are highlighted in grey. CIx—cholestatic index; CyA—cyclosporine-A; d—day; PQ—paraquat; TAR—tartrazine; TRI—triclosan.

**Table 4:** Calculated safety margin for the cholestatic risk of CyA in the three PHH donors.

	d7		d14		d21		d28	
	CyA Conc. µM *	SM_CHOL_	CyA Conc. µM *	SM_CHOL_	CyA Conc. µM *	SM_CHOL_	CyA Conc. µM *	SM_CHOL_
S1353T	5	6.50	5	6.50	5	6.50	5	6.50
S1391T	-	-	5	6.50	0.5	0.65	0.05	0.06
S1506T	5	6.50	5	6.50	5	6.50	5	6.50

* is the lowest concentration producing a CIx below 0.80 at the respective time-point. The SM_CHOL_ was calculated considering the Cmax_total_ of CyA. A SM_CHOL_ under 30 was taken as an indicator as cholestatic risk [14]. CIx—cholestatic index; CyA—cyclosporine-A; d—day; SM_CHOL_—safety margin for drug-induced cholestasis.

## Data Availability

Data is contained within the article and Supplementary Material.

## Supplementary Materials

The following are available online at https://www.mdpi.com/article/10.3390/ijms222011005/s1.
