# Supplementary material for: Primary Human Hepatocyte Spheroids as Tools to Study the Hepatotoxic Potential of Non-Pharmaceutical Chemicals
Source: Int J Mol Sci. 2021 Oct 12;22(20):11005. doi: 10.3390/ijms222011005 (PMC8537720; doi:10.3390/ijms222011005)
Supplement: Supplementary file 1 [file ijms-22-11005-s001.zip › ijms-1388106-supplementary.pdf]

Supplementary Material

Primary Human Hepatocyte Spheroids as Tools to Study the Hepatotoxic Potential of Non-Pharmaceutical Chemicals

Vânia Vilas-Boas [1,†,‡], Eva Gijbels [1,†], Kaat Leroy [1], Alanah Pieters [1], Audrey Baze [2], Céline Parmentier [2] and Mathieu Vinken [1,*]

[1] Entity of In Vitro Toxicology and Dermato-Cosmetology, Vrije Universiteit Brussel, 1090 Brussels, Belgium; vfevilasboas@gmail.com (V.V.-B.); eva.anne.gijbels@vub.be (E.G.); kaat.leroy@vub.be (K.L.); alanah.pieters@vub.be (A.P.)

[2] KaLy-Cell, 67115 Plobsheim, France; a.baze@kaly-cell.com (A.B.); c.parmentier@kaly-cell.com (C.P.)

* Correspondence: mathieu.vinken@vub.be

† These authors contributed equally to this work.

‡ Current affiliation: International Iberian Nanotechnology Laboratory, 4715-330 Braga, Portugal.

This file contains extra figures of the spheroids from the different primary human hepatocyte donors included in the study and the expression of hepatocyte differentiation markers. Images supporting bile canaliculi functionality in the 3D model are also shown. Furthermore, it contains information on the cholestatic index calculated for growing concentrations of bosentan (BOS) and macitentan (MAC) at distinct time-points, as well as the representation of the synergistic effects, or their lack, observed between the bile acid (BA) mixture and BOS or MAC. Finally, the names and functions of the genes mentioned in the manuscript are listed.

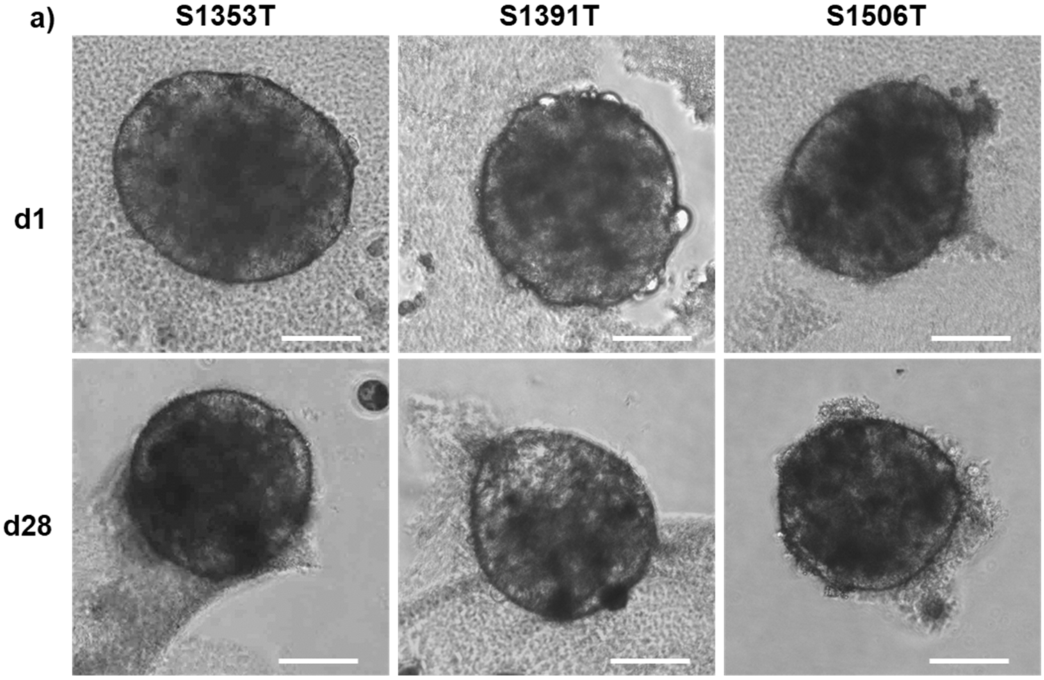

Donor	Day	ATP content (AU)	ATP content (%)	Volume (mm3)	Volume (%)	Corrected viability (%)
S1353T	1	326474	100	0.080	100	100
	28	204162	62.5	0.059	73.8	84.5
S1391T	1	275920	100	0.078	100	100
	28	171133	62	0.045	57.7	107.5
S1506T	1	201632	100	0.055	100	100
	28	113126	56.1	0.030	54.6	102.9

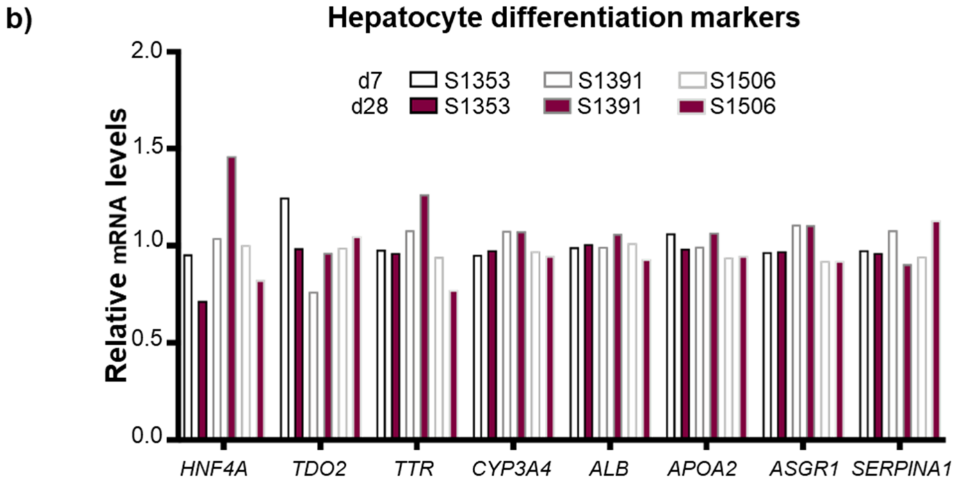

Figure S1. (**a**) Representative images of spheroids from different PHH donors, taken on day 1 and day 28 after exposure. Spheroids from different PHH donors look very similar and experience similar compaction rates throughout the time in culture. When corrected for spheroid compaction, as described in Bell et al., 2016 [1], cell viability is kept above 80% at day 28. Scale bar is 100 µm. (**b**) Expression of genes involved in hepatocyte maturation and differentiation. Transcriptomic analysis revealed that the markers were stably expressed in the long-term cultures. HNF4A—hepatocyte nuclear factor 1 alpha; TDO2—tryptophan 2,3-deoxygenase; TTR—transthyretin; CYP3A4—cytochrome P450 isoform 3A4; ALB—albumin; APOA2—apolipoprotein A2; ASGR1—asialoglycoprotein receptor 1; SERPINA1—alpha1-antitrypsin.

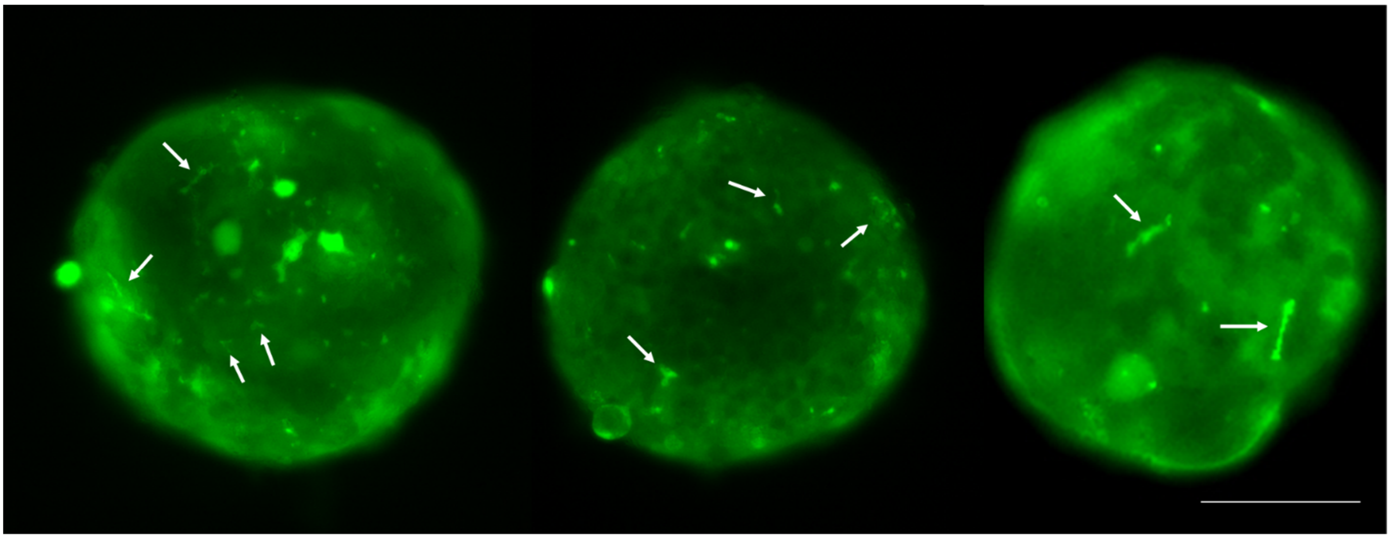

Figure S2. Primary human hepatocyte spheroids from donor S1506T stained with 5(6)-carboxy-2′,7′-dichlorofluorescein diacetate (CDFDA) supporting that the bile canaliculi are functional in the 3D model. The probe is enzymatically metabolized by viable cells to a fluorescent compound that is then transported through the multidrug resistance-associated protein-2 to the bile canaliculi, allowing the visualization of canalicular structures (white arrows) inside the spheroids. Live control spheroids collected on day 7 were stained following a protocol previously established by Tostões et al., 2012 [2]. Scale bar is 100 μm.

Table S1. Inter-donor variability regarding the effect of drug concentration and time of exposure on the cholestatic index values.

		BOSENTAN				MACITENTAN			
	μM	0.15	1.5	7.5	75	0.005	0.5	5	10
S1353T	d1	1.06 ± 0.13	0.93 ± 0.01	1.01 ± 0.11	1.01 ± 0.11	1.06 ± 0.08	1.02 ± 0.13	1.06 ± 0.00	1.00 ± 0.05
	d7	0.94 ± 0.11	0.97 ± 0.05	1.00 ± 0.11	0.93 ± 0.02	0.92 ± 0.04	0.99 ± 0.04	0.98 ± 0.01	0.99 ± 0.01
	d14	0.95 ± 0.02	0.98 ± 0.08	1.03 ± 0.12	1.03 ± 0.25	1.02 ± 0.09	1.07 ± 0.04	1.11 ± 0.03	1.08 ± 0.08
	d21	1.01 ± 0.06	0.92 ± 0.13	1.12 ± 0.18	1.38 ± 0.08	0.96 ± 0.06	0.93 ± 0.10	1.15 ± 0.15	1.25 ± 0.05
	d28	0.87 ± 0.04	1.01 ± 0.03	1.03 ± 0.04	0.42 ± 0.24	0.99 ± 0.04	0.94 ± 0.18	1.07 ± 0.20	0.95 ± 0.01
S1391T	d1	0.90 ± 0.24	1.00 ± 0.00	1.08 ± 0.05	0.91 ± 0.04	0.95 ± 0.07	0.93 ± 0.08	1.00 ± 0.13	0.94 ± 0.06
	d7	0.90 ± 0.11	0.87 ± 0.01	1.01 ± 0.16	0.92 ± 0.11	0.83 ± 0.01	0.92 ± 0.02	1.10 ± 0.11	1.09 ± 0.01
	d14	0.86 ± 0.06	0.90 ± 0.11	0.90 ± 0.09	1.1 ± 0.23	0.92 ± 0.14	0.95 ± 0.01	1.15 ± 0.25	1.10 ± 0.07
	d21	0.95 ± 0.18	1.04 ± 0.25	0.94 ± 0.04	0.70 ± 0.21	0.91 ± 0.31	0.79 ± 0.00	1.28 ± 0.25	1.20 ± 0.11
	d28	0.91 ± 0.06	1.06 ± 0.16	1.12 ± 0.30	1.12 ± 0.11	0.84 ± 0.01	0.75 ± 0.09	1.09 ± 0.14	1.05 ± 0.14
S1506T	d1	1.02 ± 0.11	0.93 ± 0.06	0.98 ± 0.06	1.03 ± 0.04	1.09 ± 0.11	1.08 ± 0.03	0.96 ± 0.05	0.91 ± 0.06
	d7	1.12 ± 0.03	0.97 ± 0.07	1.27 ± 0.15	0.98 ± 0.22	1.09 ± 0.20	1.06 ± 0.24	1.18 ± 0.00	1.29 ± 0.08
	d14	1.18 ± 0.19	1.08 ± 0.01	1.26 ± 0.11	0.79 ± 0.20	1.23 ± 0.09	1.23 ± 0.06	1.38 ± 0.08	1.34 ± 0.21
	d21	1.08 ± 0.13	0.92 ± 0.07	2.34 ± 0.21	0.45 ± 0.14	1.06 ± 0.20	1.04 ± 0.08	1.24 ± 0.12	1.27 ± 0.06
	d28	0.90 ± 0.04	1.09 ± 0.07	4.27 ± 0.28	0.43 ± 0.19	0.86 ± 0.07	1.12 ± 0.23	1.12 ± 0.25	1.13 ± 0.16

Values were calculated for each of the test compounds, bosentan and macitentan, in 4 different concentrations in the presence and absence of concentrated BA mixture, at 5 consecutive time-points, for each of the 3 donors. Data represent mean ± standard deviation values collected from at least 6 spheroids per condition. CIx values below 0.80 are highlighted in grey.

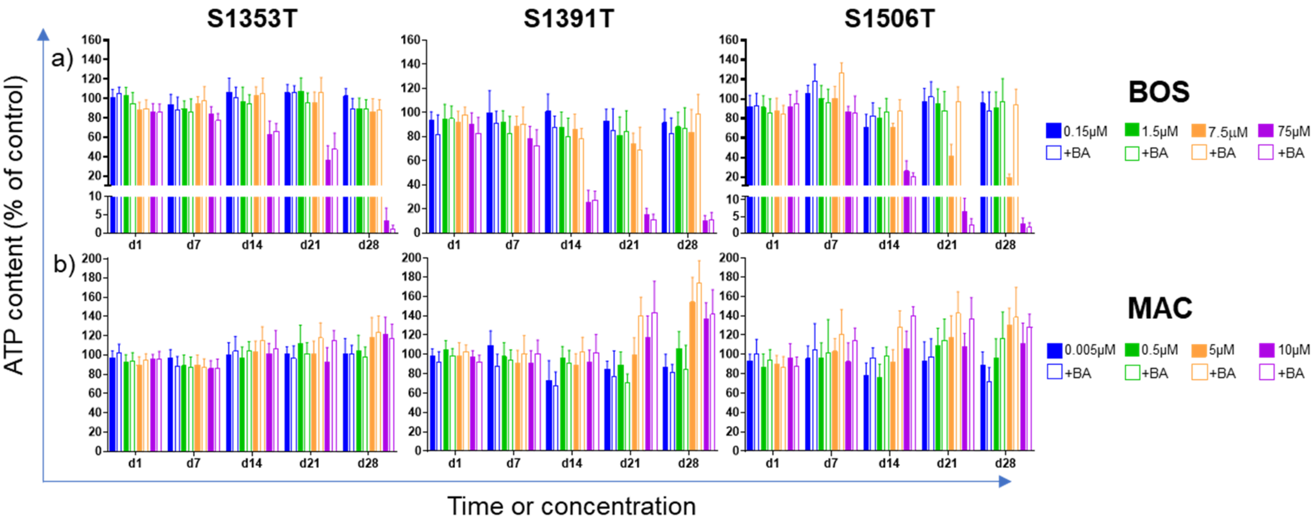

Figure S3. Effects of a concentrated BA mixture on the hepatotoxicity of the test compounds (**a**) bosentan and (**b**) macitentan throughout time. Spheroids from 3 different PHH donors were repeatedly incubated for 1, 7, 14, 21 and 28 days with the specified test compound in the presence (empty bars) and in the absence (filled bars) of a 30× concentrated mixture of BAs. At each predefined time-point, total ATP content was quantified. The effect of vehicle alone (control) was taken as 100%. Results are mean ± standard deviation of at least 6 spheroids per condition. ATP—adenosine triphosphate, BA—bile acid, BOS—bosentan, d—day, MAC—macitentan.

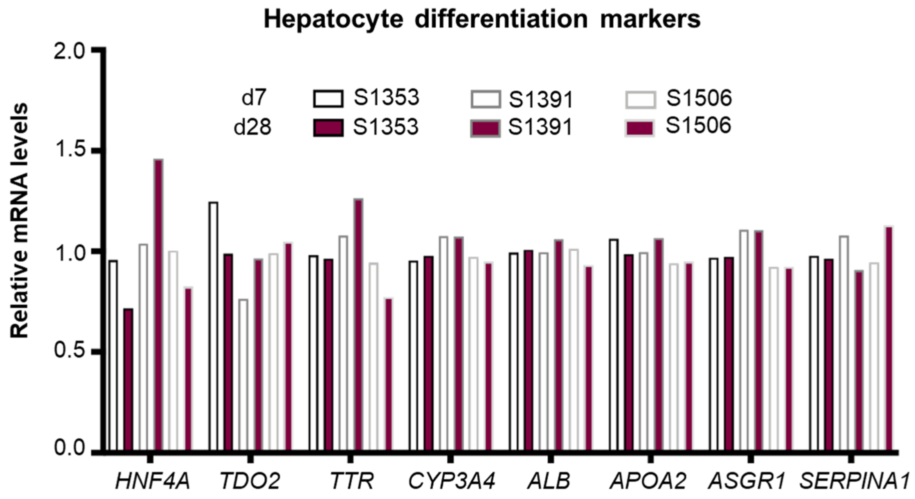

Figure S4. Expression of genes involved in hepatocyte maturation and differentiation. Transcriptomic analysis revealed that the markers were stably expressed in the long-term cultures. HNF4A—hepatocyte nuclear factor 1 alpha; TDO2—tryptophan 2,3-deoxygenase; TTR—transthyretin; CYP3A4—cytochrome P450 isoform 3A4; ALB—albumin; APOA2—apolipoprotein A2; ASGR1—asialoglycoprotein receptor 1; SERPINA1—alpha1-antitrypsin.

Table S2. Names and functions of the genes described in the analyses provided in the main manuscript.

Figure Number *	Gene Abbreviation	Gene Name	Relevant Associated Biological Pathways/Functions
4b	*CCR2*	C-C motif chemokine receptor 2	Inflammation
	CSF1	Colony stimulating factor 1	Inflammation
	IL1β	Interleukin 1β	Inflammation
	IL1R1	Interleukin 1 receptor 1	Inflammation
	MAPKAPK3	Mitogen-activated protein kinase-activated protein kinase 3	Inflammation and oxidative stress
	NLRP3	NOD-, LRR- and pyrin domain-containing protein 3	Inflammation
	SERPINE1	Serpin E1	Inflammation
	TLR3	Toll like receptor 3	Inflammation
	TNFRSF9	Tumor necrosis factor receptor superfamily 9	Adaptive immunity
	ATF4	Activating transcription factor 4	Endoplasmic reticulum stress, amino acid starvation, mitochondrial stress or oxidative stress
	ATF6	Activating transcription factor 6	Unfolded protein response during endoplasmic reticulum stress
	DDIT3	DNA damage inducible transcript 3	Endoplasmic reticulum stress
	HSPA5	Heat shock protein A5	Unfolded protein response during endoplasmic reticulum stress
	LY96	Lymphocyte antigen 96	Inflammation and apoptosis
	MAPK8	Mitogen-activated protein kinase 8	Apoptosis
	TLR4	Toll like receptor 4	Apoptosis
	TJP2	Tight junction protein 2	Apoptosis
	CASP3	Caspase 3	Apoptosis
	TP53	Tumor protein 53	Apoptosis
	KRT18	Keratin 18	Apoptosis and cytoskeletal signaling
	FAS	Fas cell surface death receptor	Apoptosis
	TNFSF10	Tumor necrosis factor superfamily 10	Apoptosis
	BAX	BCL2 associated X, apoptosis regulator	Apoptosis
	BCL2	BCL2 apoptosis regulator	Apoptosis
	ATG7	Autophagy related 7	Autophagy
	MAP1LC3B	Microtubule associated protein 1 light chain 3β	Autophagy
	SH3GLB1	SH3 domain containing GRB2 like, endophilin B1	Autophagy
	SQSTM1	Sequestosome 1	Autophagy
	CYLD	CYLD lysine 63 deubiquitinase	Necroptosis
	MLKL	Mixed lineage kinase domain like pseudokinase	Necroptosis
	RIPK1	Receptor interacting protein kinase 1	Necroptosis
	RIPK3	Receptor interacting protein kinase 3	Necroptosis
	ABCB11	Bile salt export pump (BSEP)	Apical transport of bile acids
	ABCC2	Multidrug resistance-associated protein 2	Apical transport of bile acids
	ABCC3	Multidrug resistance-associated protein 3	Alternative basolateral transport of bile acids
	ABCC4	Multidrug resistance-associated protein 4	Alternative basolateral transport of bile acids
	CYP2B6	Cytochrome P450 2B6	Drug and xenobiotic metabolism
	CYP3A4	Cytochrome P450 3A4	Drug metabolism
	CYP7A1	Cytochrome P450 7A1	Bile acid synthesis

Table S2. *Cont.*

Figure Number *	Gene Abbreviation	Gene Name	Relevant Associated Biological Pathways/Functions
4b	*NR0B2*	Small heterodimer partner	Regulation/inhibition of other nuclear receptors
	NR1H4	Farnesoid X receptor	Bile acid homeostasis
	NR1I2	Pregnane X receptor	Regulator of genes involved in metabolism and secretion of xenobiotics, drugs and endogenous compounds (e.g., CYP3A4, MDR1)
	NR1I3	Constitutive androstane receptor	Regulator of genes involved in metabolism and secretion of xenobiotics, drugs and endogenous compounds (e.g., bilirubin)
	OATP1B1	Solute carrier organic anion transporter 1B1	Transporter of endogenous compounds (e.g., bilirubin) and removal of drugs of hepatocytes
	OSTα	Organic solute transporter α	Intestinal basolateral transporter of bile acids
	OSTβ	Organic solute transporter β	Intestinal basolateral transporter of bile acids
	SLC10A1	Na^{+}/taurocholate cotransporting polypeptide	Basolateral transporter of bile acids of hepatocytes
	SULT2A1	Sulfotransferase 2A1	Metabolism of drugs and endogenous compounds, including bile acids
	UGT2B4	UDP glucuronosyltransferase 2B4	Metabolism of drugs, xenobiotics and endogenous compounds
5	*AMPD3*	Adenosine monophosphate deaminase 3	Energy metabolism and inflammation
	ADGRG1	Adhesion G protein-coupled receptor G1	Collagen binding, cell adhesion and cell-cell interactions
	BCL2L1	BCL2 like 1	Apoptosis
	CHI3L1	Chitinase 3 like 1	Inflammation and tissue remodeling and apoptosis
	CTTN	Cortactin	Organization of actin cytoskeleton and cell shape
	CXCL8	C-X-C motif chemokine ligand 8	Inflammation
	IER3	Immediate early response 3	Apoptosis
	IFI16	Interferon ɣ inducible protein 16	Inflammation
	IFI27	Interferon α inducible protein 27	Apoptosis and inflammation
	IGFBP1	Insulin like growth factor binding protein 1	Cell migration/metabolism, unfolded protein response and glucose metabolism
	IL6	Interleukin 6	Inflammation
	KYNU	Kynureninase	Metabolism of amino acids and derivates
	LDLR	Low density lipoprotein receptor	Lipid digestion, mobilization and transport
	LY96	Lymphocyte antigen 96	Inflammation and apoptosis
	MLKL	Mixed lineage kinase domain like pseudokinase	Necroptosis

Table 2. *Cont.*

Figure Number *	Gene Abbreviation	Gene Name	Relevant Associated Biological Pathways/Functions
5	*PPP1R3C*	Protein phosphatase 1 regulatory 3C	Glucose metabolism
	SDC2	Syndecan 2	Cytoskeletal organization and cell-matrix interactions
	SLCO1B3	Solute carrier organic anion transporter 1B3	Bile acid and bilirubin transport
	TFPI	Tissue factor pathway inhibitor	Collagen chain trimerization, formation of fibrin clot and hemostasis
	TNFRSF10A	Tumor necrosis factor superfamily 10A	Apoptosis
6a	*MLXIPL/CHREBP*	MLX interacting protein like	Regulator of triglyceride synthesis genes
	SREBP1c	Sterol regulatory element binding transcription factor 1, isoform c	Regulator of sterol biosynthesis genes
	FASN	Fatty acid synthase	Fatty acid biosynthesis
	SCD	Stearoyl-CoA desaturase	Fatty acid biosynthesis
	ACOX1	Acyl-CoA oxidase 1	Fatty acid β-oxidation pathway
	CD36	Cluster determinant 36 molecule	Transport of fatty acids
6b	*ACTA2*	Actin α2	Vascular contractility
	ADAMTS2	ADAM metallopeptidase with thrombospondin type 1 motif 2	Excision of fibrillar procollagens
	CDH11	Cadherin 11	Cell adhesion
	COL14A1	Collagen type XIV α1 chain	Adhesion of collagen bundles, regulation of fibrillogenesis and collagen chain trimerization
	COL15A1	Collagen type XV α1 chain	Adhere basement membranes to underlying connective tissue stroma and collagen chain trimerization
	COL1A1	Collagen type I α1 chain	Fibril-forming collagen
	COL1A2	Collagen type I α2 chain	Fibril-forming collagen
	COL3A1	Collagen type III α1 chain	Fibril-forming collagen
	COL4A1	Collagen type IV α1 chain	Collagen chain trimerization
	COL4A2	Collagen type IV α2 chain	Collagen chain trimerization
	COL4A3	Collagen type IV α3 chain	Collagen chain trimerization
	COL4A4	Collagen type IV α4 chain	Collagen chain trimerization
	COL5A1	Collagen type V α1 chain	Assembly of heterotypic fibers composed of both type I and type V collagen and collagen chain trimerization
	COL5A2	Collagen type V α2 chain	Assembly of heterotypic fibers composed of both type I and type V collagen and collagen chain trimerization
	COL6A3	Collagen type VI α3 chain	Bind extracellular matrix proteins and collagen chain trimerization
	CTGF	Cellular communication network factor 2	Cell adhesion and chondrocyte proliferation and differentiation
	CTSK	Cathepsin K	Extracellular matrix degradation, fibrinogen endoprotease activity
	DCN	Decorin	Collagen fibril assembly

Table S2. *Cont.*

Figure Number *	Gene Abbreviation	Gene Name	Relevant Associated Biological Pathways/Functions
6b	*FBN1*	Fibrilin 1	Structural component of microfibrils of the extracellular matrix
	ITGB8	Integrin β8	Mediate cell-cell and cell-extracellular matrix interactions
	MMP1	Matrix metallopeptidase 1	Degradation of extracellular matrix
	PCOLCE2	Procollagen C-endopeptidase enhancer 2	Degradation of extracellular matrix and collagen chain trimerization
	PPP1R3C	Protein phosphatase 1 regulatory 3C	Glycogen biosynthesis and cyclic AMP-dependent protein kinase A
	SPP1	Secreted phosphoprotein 1	Degradation of extracellular matrix and cell-matrix interactions
	TGF β 1	Transforming growth factor β1	Stimulation of collagen production
	TGF β 2	Transforming growth factor β2	Extracellular matrix organization
	THBS2	Thrombospondin 2	Mediate cell-to-cell and cell-to-matrix interactions
	VCAN	Versican	Cell adhesion and tissue morphogenesis and maintenance
	VWF	Von Willebrand factor	Coagulation, hemostasis and adhesion of platelets

* in the main manuscript. Information collected from Uniport.org, Genecards.org, Reactome.org and https://www.ncbi.nlm.nih.gov/biosystems.

References

1. Bell, C.C.; Hendriks, D.F.; Moro, S.M.; Ellis, E.; Walsh, J.; Renblom, A.; Fredriksson Puigvert, L.; Dankers, A.C.; Jacobs, F.; Snoeys, J.; et al. Characterization of primary human hepatocyte spheroids as a model system for drug-induced liver injury, liver function and disease. *Sci. Rep.* **2016**, *6*, 25187.
2. Tostões, R.M.; Leite, S.B.; Serra, M.; Jensen, J.; Björquist, P.; Carrondo, M.J.; Brito, C.; Alves, P.M. Human liver cell spheroids in extended perfusion bioreactor culture for repeated-dose drug testing. *Hepatology* **2012**, *55*, 1227–1236.
